# Medical News and Miscellany

**Published:** 1892-10

**Authors:** 


### Medical News and Miscellany.

Cholera is a back number now.

Dr. C. E. Dallas has removed from Forest to Alto, Texas.

Dr. C. E. Kellar has removed from Luling to Olmus, Texas.

Dr. J. C. Carpenter has removed from Buda to Kyle, Texas.

Dr. D. G. Shirley has removed from Tyler, Texas, to Mt. Sylvan, same county.

Dr. W. C. Wile has been elected Surgeon-General of the Grand Army of the Republic.

Prof. Seth M. Morris, of the Texas Medical College, returned from Berlin 20th of September.

Dr. W. G. Jameson has resigned the position of physician to the Rusk penitentiary to accept the position of surgeon to I. & G. N. R. R.

**Dr. J. O. Lewright**, of Austin, who spent the summer in Berlin, studying, returned via New York and reached Austin Sept. 27th, in fine health.

---

**Dr. W. B. West**, of Fort Worth, has associated with him in practice Dr. James Anderson, a graduate of the University of Edinburgh, and the firm will be Drs. West & Anderson.

---

**The** Governor has revoked a portion of the proclamation of April 29th, imposing restrictions on all vessels from south of 25° north latitude; but that part relating to infected ports, persons and things remains in full force.

---

**Mr. Wade Morris**, son of Dr. W. A. Morris, of Austin, who has been studying dentistry with Dr. Casper, of this city, has matriculated at the dental department University of Pennsylvania, and will spend two years there.

---

**Hymenial.**—The JOURNAL acknowledges the courtesy of an invitation to the wedding ceremony of Miss Lea Wolff, daughter of Dr. A. S. Wolff, the State Quarantine Officer at Brownsville, Texas, to Mr. Bernand L. Cain, which event will take place in Brownsville, Wednesday evening, October 26, inst.

---

**Dr. E. G. Nicholson**, of Del Rio, Texas, one of the finest men in the Texas profession, a man of splendid attainments and much beloved by all who know him, the JOURNAL is pained to announce, is a patient at the Santa Rosa hospital, San Antonio, suffering with Bright's disease, and but little if any hopes for his recovery are entertained.

---

The danger of cholera infections from New York being over, State Health Officer Swearingen has withdrawn the quarantine officers who were inspecting trains at every entrance into Texas, and closed all the stations. All the gulf stations too, were closed on the 15th inst., except Galveston, Brownsville and Sabine Pass. Sabine Pass will close November 1st.

---

**We solicit contributions** to the pages of the JOURNAL. Not a doctor of any ambition or pride in his profession but meets with cases that ought to go on record, be reported both for the benefit of the profession at large, and for statistics and future use. Send us reports of cases, your views on any medical topic, or anything appropriate, and do not be afraid of criticism. Every paper contributed will be carefully edited.

---

Notice.—The JOURNAL has a *Nedofik Lounge*, new, unused, for sale, the owner not being in the practice. Cost, laid down, $65.75; will sell for $48, cash. Splendid article, and this a rare opportunity. (Factory price, $55; freight, $10.75.)

---

The JOURNAL regrets exceedingly to learn that Dr. M. De Causey, of Seeley, Tex., met with a serious and painful accident on the 29th of September, ult. Returning home at night from visiting a patient in the country, he was thrown from his buggy—the horses having become unmanageable—and badly hurt. It is thought he was stepped upon by one of the horses, as he is hurt inwardly. The following day he was vomiting blood and had hemorrhage from the bowels at the same time. The injury may prove fatal.

Big Meeting—We learned through the papers next day that there had been held a meeting of the State Eclectic Medical Association in Austin on the 12th or 13th inst. "The Electric Medical Association" the bright young man of the Austin *Statesman* got it, both in the heading and wherever it was mentioned in his account of it. It must have been a star-chamber affair, as none of the physicians of Austin knew anything of it so far as we have been enabled to learn. We understand that a "Doctor Johnson, of San Antonio," was elected president, and that they claim to number 350 in Texas. What they did no one knows, further than is shown in a meager outline published in the daily papers. The meeting was held in the parlor at the

Driskill hotel. In reading the list of names of those present we were struck with the absence of familiar names; with the exception of Dr. W. B. Ketchum, of Palo Pinto, there was not one present of whom the JOURNAL had even ever heard before. Dr. Ketchum used to take the JOURNAL but withdrew his support—don't know why.

---

The Mississippi Medical Association is called to meet in special session at Jackson, Miss., November 1st, for the purpose of recommending to the Governor "five skilled physicians" to be appointed on the State Board of Health, and for the purpose of revising the Society's Constitution. It will be remembered they got up a little split awhile back and certain dissatisfied members "bolted" (that's the word now), went off and organized, and claimed that they alone were the genuine article. It is to be hoped a revision of the laws may bring the dissatisfied element back. Why cannot doctors live in harmony in organizations of the kind?

---

Eleventh International Congress.—As a recent notice in the JOURNAL has informed our readers, the Eleventh International Medical Congress will meet in Rome, Italy, from Sept. 24 to October 1, 1893. By an official letter dated August 22, 1892, and signed by Prof. Guido Baccelli, president, and Prof. E. Maraglina, secretary general, Dr. A. Jacobi, of New York, has been directed to form an American Sub-Committee. Its membership is not yet complete, but on it are already found beside that of the chairman, the names of Drs. Wm. Osler, of Baltimore; S. C. Busey, of Washington; N. S. Davis, of Chicago, Charles A. L. Reed, of Cincinnati; Wm. Pepper, of Philadelphia; F. Peyre Porcher, of Charleston; James Stewart, of Montreal, and Alexender J. C Skeene, of Brooklyn, N. Y. In the interest of facilitating the trip to Italy and reducing the espense, arrangements will be made with the steamship companies. According to a communication from the Central Committee, contained in a letter of the secretary general's dated September 14th, the North German Lloyd proposes to reduce the fare to Genoa by 20 per cent., and that of the return trip by ten per cent. It is expected that still more favorable terms will be secured.

